# Collaboration Variability in Autism Spectrum Disorder

**DOI:** 10.3389/fnhum.2020.559793

**Published:** 2020-10-06

**Authors:** Maria Blancas, Giovanni Maffei, Martí Sánchez-Fibla, Vasiliki Vouloutsi, Paul F. M. J. Verschure

**Affiliations:** ^1^Synthetic Perceptive Emotive Cognitive Systems (SPECS) Laboratory, Institute for Bioengineering of Catalonia (IBEC), Barcelona, Spain; ^2^Barcelona Institute of Science and Technology (BIST), Barcelona, Spain; ^3^Department of Information and Communication Technology, Pompeu Fabra University, Barcelona, Spain; ^4^Catalan Institution for Research and Advanced Studies (ICREA), Barcelona, Spain

**Keywords:** autism, prediction, collaboration, sensorimotor contingencies, neurodiversity

## Abstract

This paper addresses how impairments in prediction in young adults with autism spectrum disorder (ASD) relate to their behavior during collaboration. To assess it, we developed a task where participants play in collaboration with a synthetic agent to maximize their score. The agent’s behavior changes during the different phases of the game, requiring participants to model the agent’s sensorimotor contingencies to play collaboratively. Our results (*n* = 30, 15 per group) show differences between autistic and neurotypical individuals in their behavioral adaptation to the other partner. Contrarily, there are no differences in the self-reports of that collaboration.

## Introduction

Autism spectrum disorder (ASD) is a neurodevelopmental disorder whose main impact falls in two domains: persistent deficits in social communication and restricted, repetitive patterns of behavior or interest (*DSM-5* American Psychiatric Association, 2013). ASD has been linked to a deficit in prediction abilities and to the fact that feedback is more prominent compared to feed-forward anticipatory control (Schmitz et al., 2003; Sinha et al., 2014; Wang et al., 2014).

Recent research (Sinha et al., 2014) suggests that a prediction deficit present since early development (Prediction Impairment in Autism, PIA hypothesis, in Sinha et al., 2014) could cause the diversity of expression of the autism syndrome. This theory divides the prediction difficulties among insistence on sameness, sensory hypersensitivities, interacting with dynamic objects, theory of mind, and islands of proficiency. Insistence on sameness represents repetitive actions and thoughts, inflexible adherence to routines, resistance to change, and ritualized patterns of verbal or non-verbal behavior. Sensory hypersensitivities refer to the sensory abnormalities (like hypersensitivity to bright light) experienced by individuals in the spectrum, however, these abnormalities are not caused by abnormally enhanced sensation. Individuals in the autism spectrum also have difficulties with theory of mind (that is, inferring mental states to others and ascribing causes to observations about a person through the connection of previous with current behavior), which can cause deficit-adjusting behavior to suit different social situations. Finally, they can exhibit enhanced abilities in strongly rule-based domains (known as islands of proficiency). These domains, like mathematics, musical performance, or calendar calculations, are strongly rule-based, which minimizes uncertainty.

Individuals with ASD show attenuated top-down prior expectations, which leads them to rely more on bottom-up sensory signals. They thus experience hypersensitivity, enhanced perception and sensation, and sensory overload (Mitchell and Ropar, 2004). Consequently, this dependence on perceptual evidence merged with impairments in contextualizing sensory evidence impedes understanding actions, and predicting social intentions. Nevertheless, individuals with ASD do not show difficulties in perceiving social stimuli, but rather in using them to update internal models of social interaction, what leads to impairments in social abilities (South et al., 2012; D’Cruz et al., 2013; Robic et al., 2015).

The so-called social symptoms encompass deficits in social interaction and communication. These poor “social-specific” priors compromise their interaction with others, as ASD individuals have difficulties in coping with the uncertainty that comes with social behaviors (Chambon et al., 2017). Acting together with another partner requires considering and integrating both one’s own and the partner’s next action. This planning of cooperative actions, although less studied, is also considered an aspect of sensorimotor control (Sebanz et al., 2006).

Sensorimotor integration can be defined as the brain process allowing response to specific demands of the environment by executing voluntary motor behavior (Machado et al., 2010). Planning and executing a simple movement require sensory feedback, to effectively coordinate movement while acting. Thus, sensorimotor approaches consider perception and action as a united process. This interaction between action and perception must be highlighted in sensorimotor approaches, as they are not seen as separate processes. On the contrary, actions are conferred an integral function for perception to explain cognitive functions.

To consider an anticipatory effect as reflecting prospective sensorimotor control, an action has to differ depending on the subsequent one (Rosenbaum et al., 2013; Ansuini et al., 2015). Sensorimotor contingencies (SMCs) can be seen technically as forward models that predict the expected sensory changes given a certain set of movements. Knowledge of SMCs allows an agent to simulate potential outcomes of behavioral alternatives. Impairments in sensorimotor integration could lead to ineffective use of sensory feedback in, for example, movement correction. As a result, the individual could face difficulties in coordination and sensory reactivity.

The main brain areas associated with sensorimotor integration are the cerebellum (Paulin, 1993; Glickstein, 1998) and the basal ganglia (Nagy et al., 2006; Chukoskie et al., 2013). It is not surprising, therefore, the significant differences found in these specific areas of autistic patients. For example, previous research showed a lower number of Purkinje cells in the cerebellum (Bauman and Kemper, 2005; Amaral et al., 2008) and a decreased volume in the basal ganglia (Estes et al., 2011) in ASD individuals as compared to typically developed ones.

The cerebellum is suggested to control the anticipatory and predictive adjustments of motor programs (Koziol et al., 2012). Its pathways link sensory signals to motor areas in the brain (Glickstein, 1998), which have a pivotal role in controlling and coordinating movement (Paulin, 1993). Research on autism has provided ample evidence that the cerebellum is among the most frequently disrupted brain regions in ASD (Palmen et al., 2004; Courchesne et al., 2005), with persistent differences in volume emerging since the first 2 years of life (Hashimoto et al., 1995; Stanfield et al., 2008). Studies suggest that ASD is characterized by alterations of the brain’s inference on the causes of socially relevant signals, and this lack of ability to predict actions of other individuals stems from cerebellar dysfunctions (Schmitz et al., 2003; Sinha et al., 2014; Wang et al., 2014).

The basal ganglia play a functional role in sensory integration and motor control (Nagy et al., 2006). This area, reciprocally connected to the cerebellum (Chukoskie et al., 2013), has previously been claimed to be different in individuals with autism. For example, it has a lower volume than typical brains (Estes et al., 2011), and one of its areas, the striatum, shows larger functional connectivity in individuals with autism (Di Martino et al., 2011). Previous research has shown weak connectivity between sensory and motor brain areas in individuals with autism (Oldehinkel et al., 2019). These findings are consistent with the sensory symptoms (such as hypersensitivity) experienced in ASD. They are also in line with work showing out of sync interactions between visual and motor regions in individuals in the spectrum.

The aforementioned alterations in sensory input and motor execution could play a pivotal role in autism. The available evidence seems to suggest that autism shows widespread disturbances in sensorimotor behavior (Haswell et al., 2009; Rinehart and Mcginley, 2010; Cook et al., 2013; Gowen and Hamilton, 2013; Thompson et al., 2017). Along similar lines, self-reports about sensorimotor behavior coming from people in the spectrum provide further evidence on sensory alteration and over-responsivity (Kern et al., 2006; Ben-Sasson et al., 2009; Tavassoli et al., 2014).

Some examples of sensorimotor alterations in ASD comprise impaired motor processing and higher detection of unattended changes compared to neurotypical individuals. There is support presenting these impairments in movement and sensory responsivity not as a peripheral feature of autism, but as a fundamental cause of the social and communicative impairments seen in the condition (Leary and Hill, 1996; Hilton et al., 2007; Reynolds et al., 2011; Matsushima and Kato, 2013). Sensorimotor difficulties in autism are associated with the development and maintenance of social impairments characteristic of the disorder. Integrating sensory information from the environment is required to plan and execute movement effectively, to, altogether, carry on proper social reciprocity.

The relation between sensorimotor impairments and social deficits in autism suggests impairments in the coupling of perceptual and social cues. More specifically, ASD individuals may encounter difficulties using the sensorimotor contingencies exhibited by another agent to predict the agent’s behavior. Thus, this work focuses on the evaluation of the coupling of perceptual and social cues based on sensorimotor interaction and the ability to predict another agent’s behavior. More specifically, we aim to assess how predictive abilities affect collaborative interaction and how they differ between ASD and Typically Developed (TD) individuals.

To do so, we devised a predictive game task where participants collaborate with a synthetic agent that displays different behavioral patterns expressed through sensorimotor contingencies. The proposed task is an adaptation of the game of Pong, where players in collaboration with a synthetic agent need to intercept a falling target (see the following section for more information). To succeed in this task, players need to identify and learn the social characteristics of the agent. Doing so will allow them to use this information during the interaction, and to later adapt more efficiently when the task becomes uncertain. As the agent’s behavior is based on sensorimotor information, we hypothesize that ASD individuals will show deficits in successful social predictive/anticipatory skills. To assess the differences in prediction between ASD and TD players, we look at aspects of adaptive collaborative skills by analyzing the interaction of the players with the AI agent of the game, how the interaction evolves during the task, and how it relates to the participants’ understanding of the other agent’s characteristics. More specifically, we study partner monitoring and how it affects the covered space and look at the mutual influence between the player and the AI-controlled agent.

We hypothesize that:

• Participants in the autism spectrum will show slower and less adaptation to the other agent than neurotypical ones.
• Participants in the autism spectrum will show less adaptation to the other agent when the task becomes more uncertain.
• Participants in the autism spectrum will show more variable behavior than neurotypicals.

## The Scenario

### The Task

**FIGURE 1:**
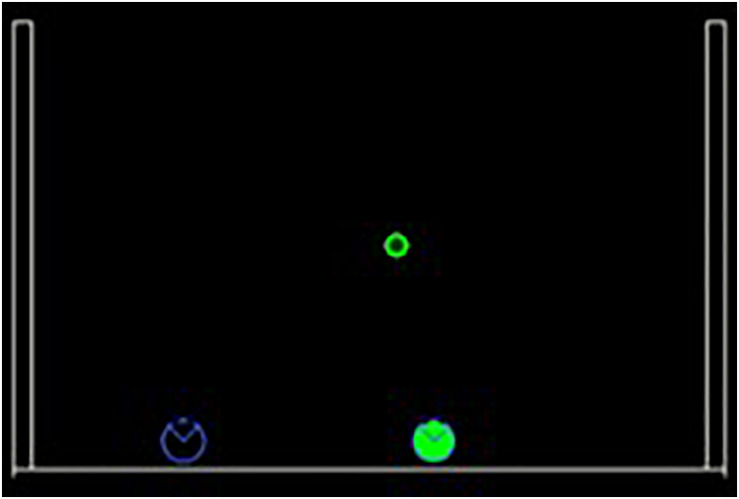
Example of a trial during the task. Targets fall from the top of the screen and players need to intercept them before they hit the ground. The player on the left (blue) is controlled by a human and the one on the right (green) is the synthetic agent. This example represents interaction with the “Middle” agent.

The purpose of the current study is to evaluate how goal-oriented coordination between partners could be achieved through sensorimotor adaptation. To do so, we designed a collaborative multiplayer version of the game of Pong: a computer version of a 2D tennis where two players try to intercept falling targets from the top before they hit the ground by moving their paddles at the bottom of the screen. The paddles move on the same horizontal line and can push each other but cannot switch sides. In this game, one player is AI-controlled, and the other is a human. Figure 1 represents an example of the proposed scenario.

For this task, we considered a collaborative team task like playing tennis doubles, where each player should cover a maximal part of their field so that all targets return to the opponent’s side. Targets sometimes fall in the middle part of the field, thus in a zone where both players could intercept the target. The location of the target was randomly selected from a uniform distribution of possible angles, and the pace of the target drop was uniform across all trials. The velocity of the artificial player was controlled and the same across all trials and the velocity of the participant depended on their motion on the trackpad. A player can be characterized by the area they cover and intervene, given the target’s direction. Typically, in a game of two, the area covered by each player is half of the playable area. However, more active players may sometimes overpass their area to try and catch ambiguous targets directed toward the middle area. Collisions with the other agent were penalized by subtracting a point, and participants were informed about the penalty before beginning the task. To evaluate whether the synthetic agent’s behavior and predictability can influence the humans’ behavior, we varied the playing styles of the agent.

The AI-controlled player differs in the way it approaches the target and the area in which it will intervene, resulting in three different agents: “Wider,” “Narrower,” and “Middle.” A “Middle” agent will try to intercept any targets that fall within its half of the space and has a 0.5 probability of intercepting an ambiguous target that falls in the middle. A “Wider” agent will try to intercept the target and overpass its area to try and catch a target even if the target’s position is not ambiguous. In contrast, a “Narrower” agent would try to intercept the target without overpassing its area; in fact, it would cover a space that is smaller than half of the overall space. The next section explains in more detail how the agent’s behavior is obtained.

### The Point of Social Subjective Equality

**FIGURE 2:**
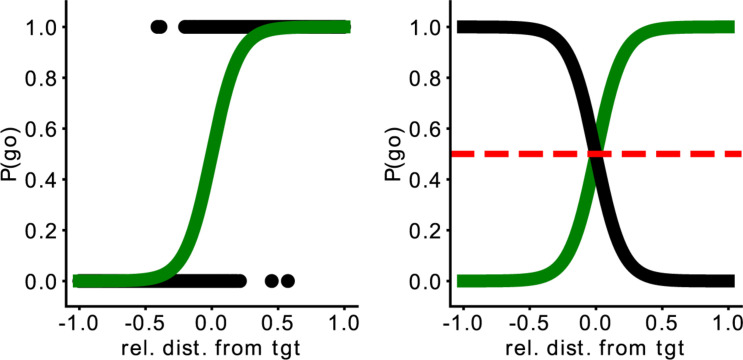
The left image represents the probability to go for the target depending on the player’s relative distance to the target. The right image depicts the Point of Social Subjective Equality (PSSE). Here, the green line represents the AI agent (in this case, the “Middle” one) and the black line, a simulated perfectly matched participant. The red dashed line represents the moment when both agents have the same probability of going for the target. The x-axis represents the relative distance of the agent from the target; the y-axis, the probability it has to go for the target.

To measure the collaboration between the human player and the AI player, we introduce the Point of Social Subjective Equality (PSSE). The PSSE can be computed for every two players and all possible target trajectories. This measurement is an analytical measure of collaboration (i.e., social affordance gradient) that defines the probability of going for the target depending on the target’s position (Figure 2, left). Therefore, the PSSE is the point where each player has the same probability of going to intercept the target (Figure 2, right) and is an extension of the Point of Subjective Equality (PSE) (Stoloff et al., 2011) to a socially collaborative task. PSE represents the point where there is an equal probability of using any of the two hands to reach a target (presented from left to right circularly in front of the participant). Thus, the Point of Social Subjective Equality indicates how a player is relying or not on the partner, invading or not the partner’s area of the field while intercepting targets in the horizontal range. In short, it is the point where a player has an equal probability of intercepting the target or letting their partner intercept it. To calculate it, we first calculate the relative distance from the player to the target (Eq. 1), that is, the difference between one player to the target and the other player to the target. After that, we fit a sigmoid function with the distance to the target (*rel_dist*), a constant factor (*k*), and a bias value (*b*) representing the behavior of each of the agents (Eq. 2). We estimated the parameters of the PSSE (*k*, *bias*, and *rel_dist*) by running a logistic regression using sklearn^1^. To our knowledge, this is the first time such a direct behavioral measure of collaboration is introduced.

(1) $$r\,e\,l\,\_\,d\,i\,s\,t=\frac{\left(\left|p_{t}-p_{p\,2}\right|-\left|p_{t}-p_{p\,1}\right|\right)}{w}$$

Representation of the relative distance (rel_dist). *p*_*t*_represents the position of the target, *p*_p2_ represents the position of the other agent, *p*_p1_ represents the position of the participant, and *w* represents the width of the (game) screen.

(2) $$P\,S\,S\,E=\frac{1}{1+e^{-\left(k\times r\,e\,l\,\_\,d\,i\,s\,t+b\right)}}$$

Representation of the Point of Social Subjective Equality (PSSE). *b* represents each partner’s bias, *rel_dist* is the relative distance from the target, and *k* is a constant factor (*k* = 20).

**FIGURE 3:**
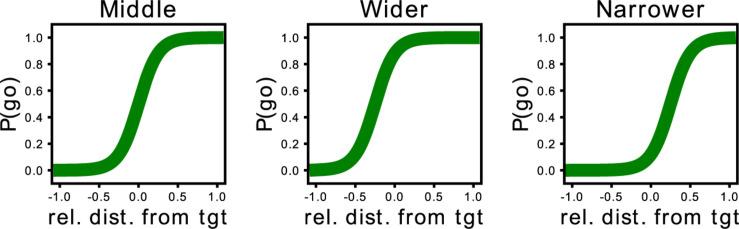
Representation of the curve of the probability to intercept the target based on the target’s location for each of the three proposed agents. From left to right: the curve of the “Middle” agent lies in the middle as both the synthetic agent and the human player have the same probability of intercepting the target. In contrast, the curve of the “Wider” synthetic agent is slightly skewed toward the left, as this agent will enter the space of the human participant. In contrast, the “Narrower” agent’s curve is skewed toward the right; this agent has a higher probability of staying toward its half of the space and allowing the human participant to intercept the target.

Based on the PSSE, two complementary partners would intercept the target with the same probability (*P* = 0.5, Figure 2, right), whereas any shift would indicate a lack of balance between the partners. As mentioned previously, participants play with three different AI agents, and we modulated their behavior based on this shift of the interception point. Our three proposed agents, namely “Middle” (M), “Wider” (W), and “Narrower” (N), have therefore different probabilities of intercepting the target. More specifically, the “Middle” agent has a 0.5 probability of going for an ambiguous target (when the target falls in the center of the arena). A “Wider” agent is more prone to invade the space of the participant; therefore, the curve of the probability to intercept the target based on the target’s location would fall toward the left part of the space. In contrast, the “Narrower” agent is more prone to stay in its half of the space and allow the participant to enter the AI agent’s space to catch the target. Consequently, the curve would fall toward the right part of the space. Thus, if we split the playable area into two equally sized sides, one for the participant and the other for the synthetic agent, a “Middle” agent would cover only its 50% of the space, while the “Wider” would cover more than 50% and the “Narrower,” less. Figure 3 provides an example of the representation of the curve for each AI agent. The agents were programmed to catch the target following a pre-defined strategy (M, W, or N). Consequently, if a participant decided to leave the target to the artificial agent, the agent’s behavior would depend on the predefined strategy and therefore the position of the target and the relative positions of the two players. Thus, there would be cases where the ball would be intercepted by the artificial agent and others where it would be missed, however, the PSSE sigmoid function would not be affected by the movement of the human player. The coefficient and the intercept of this curve will allow us to assess participants’ adaptation to the other agent.

## Materials and Methods

### Participants

The ASD participants recruited for the study had previously been diagnosed as autistic, meeting the *DSM-5* criteria for level 1 of autism (“Requiring support;” American Psychiatric Association, 2013) (*N* = 15, one female, age: 18.67 ± 2.4). This criterion comprises difficulties in initiating social interactions and switching between activities. This group was recruited in the Educa Friends center^2^, an educational support service part of the Friends Foundation, focused on providing support to high-level functioning ASD individuals. All participants had a normal or corrected-to-normal vision and were not color blind. Participants were matched for handedness (all of them were right-handed) and almost matched for age (same mean, different standard deviation) and gender (only one female more in the TD group). The typically developed participants were recruited in a high school of Barcelona and the campus of the Polytechnic University of Catalunya, and their age matched those with ASD (*N* = 15, two females, age: 18.38 ± 1.06). Written informed consent was obtained for all participants (for the ones under the age of 18, parental written informed consent was obtained too). The study was approved by the local ethical committee (Parc de Salut del Mar).

### Apparatus

**FIGURE 4:**
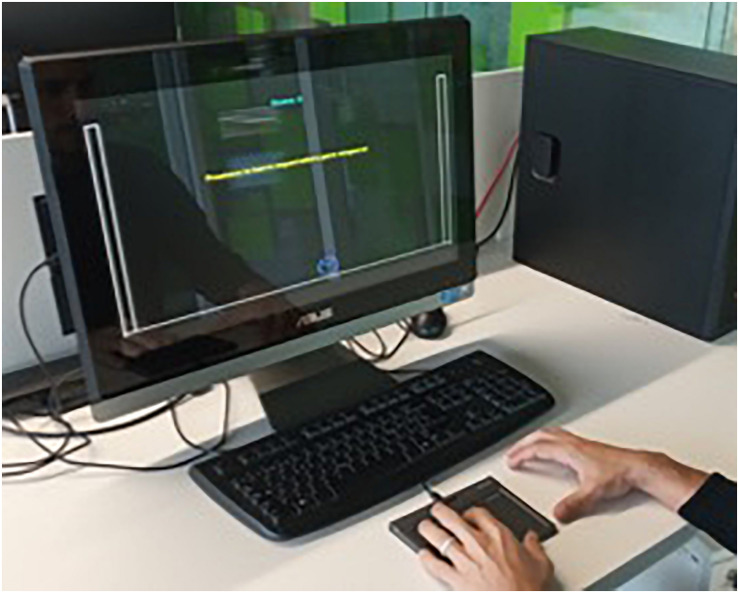
Representation of the setup used in the task. Participants sit in front of a computer screen where the game was displayed. Participants controlled the motion of their avatar using a touchpad.

Participants sat at a viewing distance of (approximately) 50 cm from a 27-inch monitor that operated at a resolution of 1,920 × 1,080. The monitor was part of an All-in-One desktop computer connected to a touchpad and a keyboard. The task was generated using Python and the PyGame library, and participants controlled their avatar using the touchpad. There was no auditory feedback during the task. Figure 4 depicts the setup used.

### Experimental Procedure

All participants were provided with an information sheet that contained the explanation of the task and a consent form they had to sign before beginning the experiment. For the underaged participants, information sheets and consent forms were given to both participants and their parents/legal tutors. Before the main task, participants filled in a small questionnaire with demographics and the frequency of playing video games. As mentioned earlier, the task is a Pong adaptation, and the goal is to intercept falling targets. The task was performed in a computer using a touchpad and consisted of three main phases. In “Phase 1,” participants played alone for one block. In all phases, each block consisted of 150 trials. The number of trials was decided after running a pilot study with other participants (excluded from this sample), both in the spectrum and neurotypical. To our understanding, 150 trials are enough to cover the probability distribution for each participant, as players do not really cover all the horizontal range of the screen. According to the design of the task, both players go for the target within their field and only need to decide whether to go or not when the target falls in the middle. Thus, the focus is on the center of the distribution, narrowing the range of interest by requiring fewer samples to build a probability distribution for each participant. In “Phase 2,” participants played for one block with each of the three AI players (in total three blocks), and finally, in “Phase 3,” participants played for one block with all agents. The order of the three AI players in “Phase 2” was randomized and for each type of the three AIs. “Phase 3” was used to assess the social predictive abilities of the participants, as they had to interact with a random agent in every trial (counterbalanced so there were 50 trials with each agent). Each of the three agents was depicted in a different color (Neutral, blue; Wider, green; and Shy, White). Color choices were made arbitrarily. Players’ positions were initialized to the center of their side at the beginning of each trial. Participants were instructed to avoid hitting the other agent and were penalized with one point less if they did.

**FIGURE 5:**
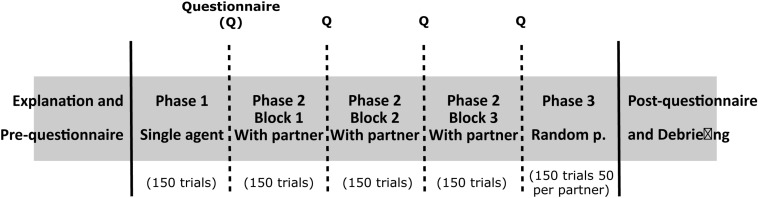
Representation of the experimental protocol. First, participants are introduced to the task and fill in a short questionnaire. The task comprises three phases, which contain one, three, and one blocks (of 150 trials per block), respectively. In “Phase 1,” participants play alone. In “Phase 2,” participants randomly play with each of the AI agents for one block (three blocks in total). In “Phase 3,” participants are presented with a random agent in each trial (50 trials per agent). Self-reports on perceived collaboration, engagement, and agents’ and target predictability are presented between phases/blocks. Finally, participants fill in a short questionnaire and undergo an interview and debriefing.

In this study, we used behavioral data, questionnaires, and interviews as instruments to collect information about the participants’ behavior and perception of the task. Between each of the blocks, participants had to answer questions in a tablet. The questions involved perceived collaboration and predictability of the target and the other agent and engagement. To answer, participants had to rate each of them on a Likert scale from 1 to 5. At the end of the task, we carried out a semi-structured interview to assess the perceived differences between agents, followed by a debriefing session. Figure 5 represents the experimental protocol. In total, the whole experiment took around 30 min and was conducted in Spanish or Catalan, depending on the preferred language of the participant.

### Data Collection

To evaluate the behavioral and perceptual differences between the two groups, we collected data gathered from the logs of the game (behavioral), questionnaires, and short interviews (perceptual). More specifically, from the logs of the game, we obtained in a trial-by-trial basis the *performance* (one point if either the participant or artificial player intercepted the target), the *identifier* of the artificial player (M, W, N), and *the position of the player, target, and agent*. The last three positions (the positions of both agents during the last three time frames in the trial) allowed us to obtain the *PSSE* measure (by analyzing their relative distance when one of them intercepted the target), as explained in Eq. 1.

The between blocks questionnaire allowed us to assess participants’ perception of the task and the artificial agent. In all blocks, participants evaluated *task engagement* and *target predictability*. In “Phase 2” and “Phase 3,” where the artificial player was introduced, participants also evaluated the agent’s *predictability* and *collaboration*. All items were reported on a 5-point Likert scale. At the end of the experiment, participants were also asked to report if they thought the other player was a human or a computer, adding “I do not know” as a possible answer.

Finally, the short-structured interview at the end of the experiment allowed us to assess with further detail participants’ perception of the task and the other agents. More specifically, we asked participants to report on the overall perceived difficulty of the task and report on how they perceived the other player. Here, participants could describe the other player and if they have identified any differences between the blocks. Furthermore, we asked participants to assess the difficulty of “Phase 3” of the task (in each trial, participants played with a random AI) and report if they followed any strategy.

## Results

The following results have been analyzed in Python, using the following libraries: NumPy, JSON, math, scipy, and sklearn. In order to choose the statistical tests used in this analysis, we ran normality tests in the variables. The intercepts of the PSSEs during “Phase 2” had a normal distribution, so parametric tests were used (One-Way ANOVA, in this case). The mean squared errors in “Phase 2” did not show a normal distribution, so non-parametric tests were used (Mann-Whitney U).

### Behavioral Results

We defined performance as the ratio of caught targets out of the 150 of each phase. A Mann Whitney *U* test (*U* = 892448.0, *p* = 0.433) showed that there were no significant differences between the two groups (ASD: median: 1.0, MAD: 0; TD: median: 1.0, MAD: 0) in *performance* during “Phase 1” (when participants played alone). Thus, possible differences in “Phase 2” and “Phase 3” should not be related to their performance when playing alone.

### Participants in the Autism Spectrum Showed More Variable Behavior Than Neurotypicals

**FIGURE 6:**
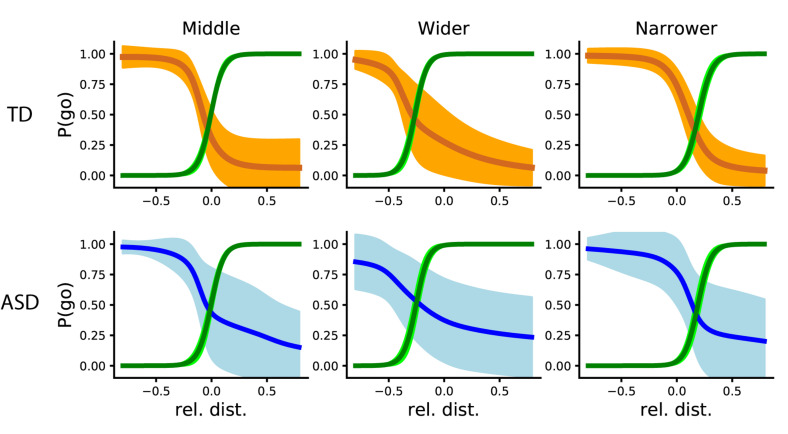
PSSE intersections from the TD and ASD groups (top and orange and bottom and blue, respectively). From left to right: the PSSE curve of the AI agent (depicted in green) and that of the participants, depicted in orange (TD) and blue (ASD), when playing with a “Middle” (N), “Wider” (A), and “Narrower” (S) agent respectively. The bright colors represent standard deviation, while the darker and thicker line represents the mean.

To assess participants’ adaptation to the artificial player, we calculated the Point of Social Subjective Equality (PSSE). First, we look at the two groups’ behavior in “Phase 2,” where we take into account all trials with each agent per block. To analyze the differences between groups and agents, we calculated the differences between the coefficients among groups for the same agent, and among agents for the same group. There were no differences between agents in their coefficients in none of the groups (Figure 6). In terms of intercepts, there were significant differences between agents in both the ASD [One-way ANOVA (6,749) = 5.68, *p* = 0.007] and TD group [One-way ANOVA (6,749) = 10.83, *p* < 0.001]. More specifically, an independent samples *t*-test showed differences in the ASD group were between the Middle and Narrow agents [*t*(4,499) = −2.11, *p* = 0.04] and the Narrow and Wider agents [*t*(4,499) = 3.46, *p* = 0.002]; and in the TD between the Middle and Narrow agents [*t*(4,499) = −5.17, *p* < 0.001] and the Narrow and Wider agents [*t*(4,499) = 4.01, *p* < 0.001].

The lack of significant difference between slopes could mean that, generally, both groups adapted in a similar way. Nevertheless, as we can see in Figure 6, participants in the ASD group showed a higher probability of going (∼0.25) with a relative distance larger than 0. This means that they had more tendency to go toward the target than the TD group (which probability at that time was around 0.07), even when they should not. The differences in intercept represent the adaptation of each group to the specific agent they were playing with. In the next sections, we will quantify the variability of each group and their behavioral changes with respect to the other agent.

To assess the variability among participants in each group, we calculated the mean squared error between each participant and the general mean. To do so, we first calculated the general mean of the coefficients extracted from the data points obtained in all trials in all blocks from “Phase 2” in both groups. From that, we calculated the average of those data points and obtained a representative mean squared error per group (ASD: 0.08 ± 0.08; TD: 0.001 ± 0.001). A Mann Whitney *U* test was used to analyze the differences between groups against the general mean (*U* = 9.0, *p* < 0.001). Moreover, when assessing the variability inside of each group (that is, the variability compared to the mean of their group), the difference is even higher (*U* = 0, *p* < 0.001). The U equal to zero signifies that all the mean squared errors in the ASD group are greater compared to all the ones in the TD group.

### Participants in the Autism Spectrum Showed Slower Adaptation to the Artificial Agent Than Neurotypical Ones

**FIGURE 7:**
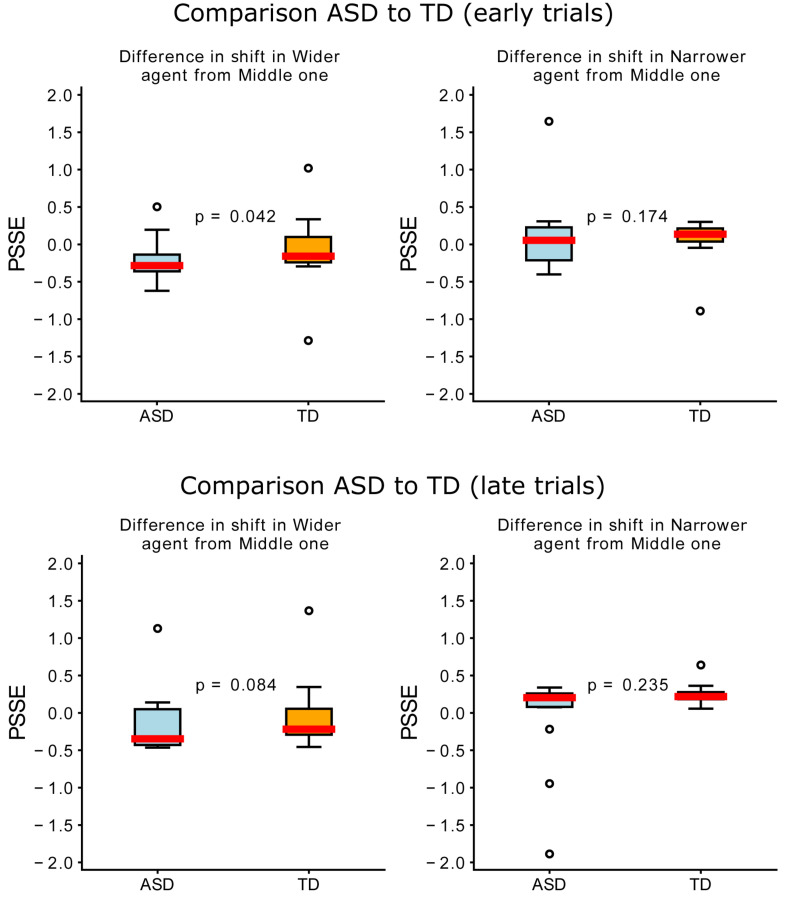
Differences between groups in the difference in shift per agent (compared to the “Middle” one). The upper plots represent early (0–50) trials and the lower plots, late (100–150) trials. Blue represents the ASD group; orange, the TD; and the red line represents the median.

To further understand the two groups’ adaptation, we then looked at a possible evolution in time of the PSSE, and more specifically, whether early (50 first trials) and late (50 last trials) trials differed between the groups in “Phase 2.” To do so, we analyzed the shift in PSSE for each of the agents. We used the “Middle” agent as a baseline and subtracted from it the shift for the “Wider” and “Narrower” agents. Like this, we could calculate how much the participants’ behavior changed when encountering the “Wider” and “Narrower” agents. As we can observe in Figure 7, we found statistically significant differences between the two groups for the “Wider” agent in the early trials (*U* = 65.0, *p* = 0.042) but not the late trials. We did not find any statistically significant differences between groups for the “Narrower” agent in both early and late trials.

### Participants in the Autism Spectrum Showed Less Adaptation to the Other Agent When the Task Became More Uncertain

During “Phase 2” we have shown that healthy subjects acquired an *ad hoc* behavioral strategy (i.e., PSSE shift) from the interaction with each individual agent and that the adaptation process was more pronounced in healthy subjects compared to control. During “Phase 3” we aim at assessing whether this strategy can be correctly retrieved when the subjects interact with each agent in a randomized order. We hypothesized that the ASD group will be less able to retrieve a correct strategy, potentially due to the reduced ability to form an internal model of the partner. To do so, for every subject we compute the PSSE associated to each agent during “Phase 2.”

In “Phase 2,” participants played for one block with each of the three AI agents. In contrast, in “Phase 3,” participants encountered a random AI player in each trial for one block. As mentioned earlier, the characteristic that distinguishes the agents’ behavior is the color, and if players have not made the color association with the agent’s behavior, “Phase 3” becomes more uncertain. Here, we wanted to assess how much the players’ behavior in “Phase 3” matches that of “Phase 2” when playing with the same agent during each of the blocks. To do so, we ran a logistic regression using participants’ behavior during “Phase 2” as our “training data,” and compared against their behavior during Phase 3, which was used as “testing data.” PSSE for each agent is described by a logistic function with constant k and intercept i. We further group the trials from “Phase 3” according to the agent type and extract, similarly to “Phase 2,” the probability of the subject to go for the target or to let the partner go (*p* = 1 and *p* = 0, respectively). Finally, we compute for every agent how accurately the parameters of the PSSEs from “Phase 2” describe the behavior (i.e., probability of going for the target) observed in “Phase 3.” The rationale is that high accuracy of the model from “Phase 2” in describing the behavior of “Phase 3” would confirm the hypothesis that a behavioral strategy tight to each individual agent has been learned and can be correctly retrieved.

**FIGURE 8:**
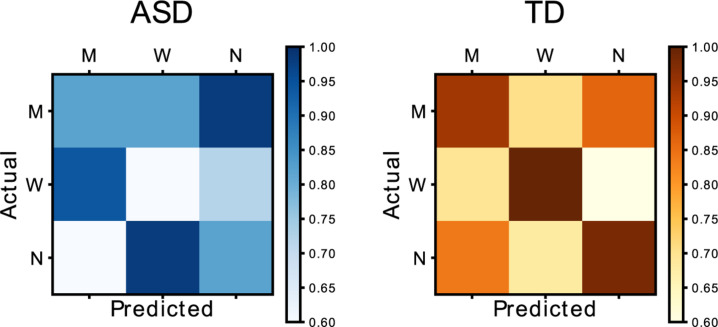
Matrix representing the relationship between Phase 2 (actual) and Phase 3 (predicted) behavior. The blue matrix represents the group in the autistic spectrum, while the orange one represents data from the neurotypical group.

In the left panel of Figure 8, we show the mean accuracy matrix for the control group and the ASD group. This is obtained by computing for every subject the accuracy of each PSSE agent model (predicted) in describing the data of each agent during “Phase 3.” This generates a set of 3 × 3 matrices that are further averaged for each group. This result suggests that the participants in the neurotypical group (right, orange) behaved in the same way in both phases, with a mean accuracy score of 0.97). However, the participants in the ASD group (left, blue) did worse in properly matching their behavior to the one in the previous phase (mean accuracy score of 0.74). A Mann Whitney-*U* test showed significant differences between the accuracy for both groups in matching Phases 2 and 3 behavior (*U* = 0, *p* = 0.03). These results could suggest that participants in the TD group developed a model of the other player during “Phase 2” that they later used to adapt their behavior in “Phase 3”; participants in the ASD group failed to do so.

### No Differences in Perception of the Task Between Groups, Only by Perceiving the Other Agent as Human or Synthetic

**FIGURE 9:**
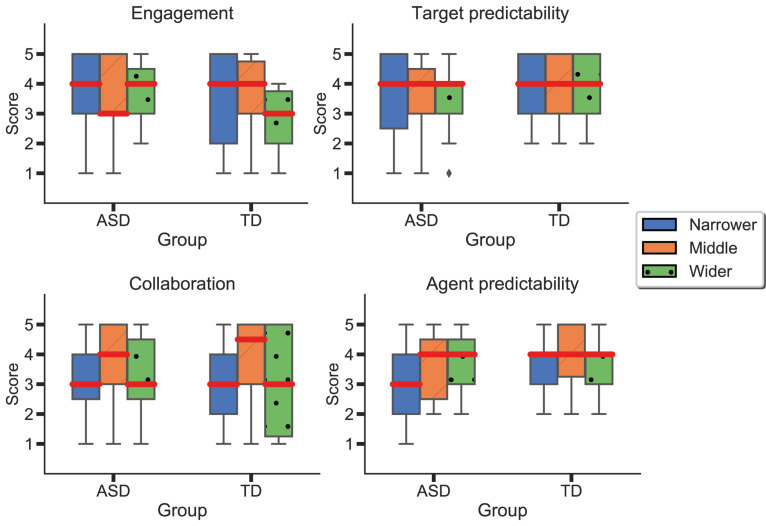
Differences between agents and groups in perceived agent predictability, target predictability, engagement, and collaboration. Blue represents the results for the “Narrower” agent; orange, for the “Middle” agent; and green, from the “Wider” one. Red lines represent means.

As previously mentioned, participants had to answer a short questionnaire between blocks. More specifically, participants evaluated target predictability, engagement (in all blocks), as well as agent predictability and collaboration (in the blocks where the AI agent was introduced). There were no significant differences in engagement or target predictability between “Phase 1” and the rest of the task. In “Phase 2,” participants rated each agent at the end of each respective block (Figure 9). Results suggest no statistically significant differences between groups in any of the dimensions. Nonetheless, participants in the ASD group seemed to feel more engaged with the task than the neurotypical group. Additionally, we observe higher variability in the ASD group when evaluating target predictability. In contrast, the TD group evaluated the target’s predictability similarly in all three blocks. Regarding collaboration, both groups reported the “Middle” agent as the most collaborative one. Finally, we could observe differences in the perceived agent predictability, where the “Narrower” agent was perceived as less predictable by the ASD group than the TD one.

**FIGURE 10:**
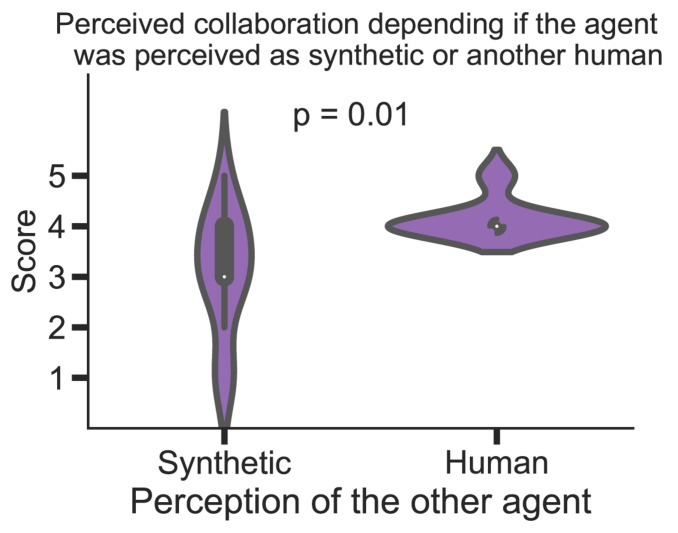
Differences in perceived collaboration between the participants that perceived the AI agent as synthetic or as a human player. The white dot represents the median.

Finally, at the end of “Phase 3,” participants reported if they were interacting with a human or a computer. There were no significant differences between the ASD and TD groups as to how many participants thought they were playing with a human or a computer. Interestingly, if we divide participants into two new groups (those that thought the other agent was a human and those who thought it was a computer), we observe differences in perceived collaboration (Figure 10). Participants that thought the other agent was a human perceived it as significantly more collaborative (Human: median 4.0, MAD: 0.0; Synthetic: median: 3.0, MAD: 1.0; Mann-Whitney U: 37.0, *p* = 0.01). More specifically, when comparing among agents (by assessing the answers during Phase 2, where participants provided self-reports for each of the agents separately), the agent that was perceived as more collaborative was the “Middle” (Human: median 5.0, MAD: 0; Computer: median: 3.0, MAD: 2.0; Mann-Whitney U: 33.0, *p* = 0.009), followed by the “Wider” (Human: median 4.0, MAD: 1.0; Computer: median: 3.0, MAD: 1.0; Mann-Whitney U: 43.0, *p* = 0.04) and the “Narrower” (Human: median 4.0, MAD: 1.0; Computer: median: 3.0, MAD: 2.0; Mann-Whitney U: 43.5, *p* = 0.04).

### Participants in the ASD Group Focused on Movement to Describe the Other Agent

**FIGURE 11:**
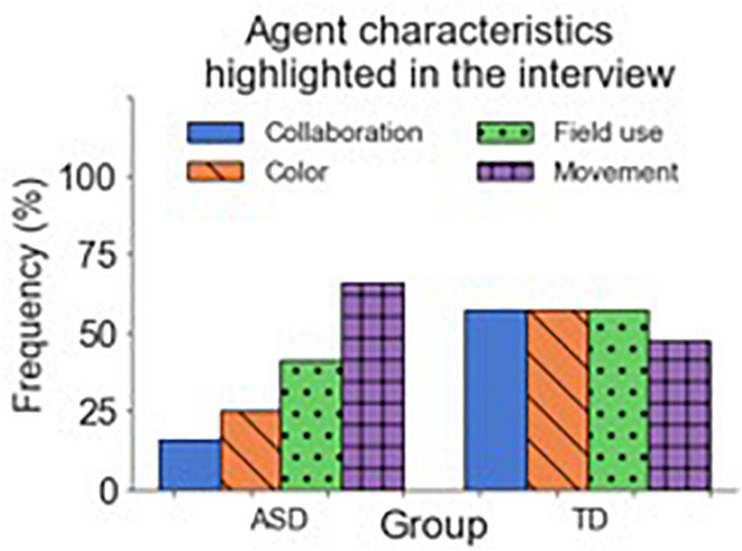
Frequency of characteristics commented about during the interviews. Blue represents collaboration-related characteristics; purple, color-related ones; green represents characteristics related to field use; and orange, characteristics related to movement. The sum of the frequencies inside each group surpasses 100% because some subjects highlighted more than one characteristic.

After the last questionnaire in “Phase 3,” participants underwent a short structured interview, which lasted ∼10 min. First, participants were asked to report the difficulty of the task and how well they performed. We later asked them to comment and describe the other agent they interacted with. In the case where participants reported differences between the agents, we also asked them to provide a short description for each agent. When participants described the agent(s) they interacted with, we identified the following common features: movement (how fast/slow the agent was perceived), field use (how much of the field the agent was using), color (the color of the agent), and collaboration (how collaborative the agent was perceived). Figure 11 depicts the frequency of use of these characteristics to differentiate between the agents (sometimes, more than one per subject). In the ASD group, the most commented characteristic was the agents’ movements (53%), followed by their field use (33%), perceived collaboration (13%), and color (6%). In the TD group, the frequency of use of the characteristics is more homogeneous. Here the most frequent characteristics are collaboration and field use (38%) followed by color and movement (30%). Moreover, one participant in the TD group differentiated between the agents by their perceived performance.

The two groups mainly differed in the type of characteristics they used to describe the other agent. Participants in the ASD used more personality-related terms to describe the behavior of the other agent (“it’s Narrower,” “it’s more selfish”) than neurotypical participants, who used a more performance-related vocabulary (“it was playing well,” “it was taking my targets”). When asked about “Phase 3,” in which they played with a random AI agent in each trial, participants in the ASD group communicated an added difficulty caused by larger uncertainty. Some reported not knowing if the other agent would go or not for the target; others reported that the task required more focus (“You never know what can happen or how will the other player react. You had to be more focused”). Only one subject reported a relationship between the agent’s color and its behavior and using it to decide to go or not for the target. In contrast, in the TD group, more participants reported using color to identify the agent and act accordingly. For both groups, “Phase 3” was perceived as more complicated and confusing than the others.

## Discussion and Conclusion

The main purpose of this study was to evaluate the ability to predict another agent’s behavior based on sensorimotor interaction and how these predictive abilities affect collaborative interaction and differ between ASD and Typically Developed (TD) individuals. We created a task where participants had to learn the behavioral characteristics (as exhibited by sensorimotor contingencies) of a synthetic agent and collaborate with the agent to maximize reward. Each player controlled an avatar, and the goal was to intercept falling targets. To assess collaboration, we developed the Point of Social Subjective Equality (PSSE) that calculates the probability of a player of going for the target given the target’s position. Finally, we examined possible perceptual differences regarding the task between the two groups.

### Discussion on Differences in Behavior

As we observe larger individual differences between participants in the autism spectrum (compared to neurotypicals), we hypothesized that participants in the autism spectrum would show more variable behavior than neurotypicals during the task. Our analysis of the differences in variability between the ASD and TD groups suggests that, indeed, the ASD group showed larger variability compared to the TD individuals.

Social impairments associated with sensorimotor difficulties are a characteristic of the disorder, and we assumed that ASD individuals would encounter difficulties in predicting the AI player’s behavior. Thus, we hypothesized that participants in the autism spectrum would show slower and less adaptation to the other agent than neurotypical ones. To assess this, we analyzed the differences between groups in adaptation to the other agent during Phase 2, and we showed differences in adaptation between groups in early trials but not in late ones, showing differences in adaptation timing. Our results show differences in the behavior of neurotypical and ASD individuals when playing with the three different synthetic agents. We observe the ability to converge to a complementary PSSE in the case of the control group. However, we do not observe the same with the ASD participants. Furthermore, we assessed the online adaptation to the artificial player by looking at the differences in errors between early and late trials among groups. Our results seem to reflect a more accurate adaptation in the neurotypical group than in the ASD.

Finally, as ASD individuals seem to find difficulties when a task is uncertain, we postulated that they would fail to predict the behavior of the AI agent correctly and, therefore, adapt less to the AI agent compared to the typically developed group. By comparing the participants’ behavior with each agent in “Phase 2” and “Phase 3,” we can assess whether they applied previously acquired information from the sensorimotor contingencies of the AI agent (“Phase 2”) to a more uncertain task (“Phase 3”) and predict the agent’s behavior. Our results suggest differences in the prediction of the agent’s behavior. More specifically, TD individuals were able to develop a better model of the artificial player in “Phase 2” and apply that information to adapt their behavior in “Phase 3,” while participants in the ASD failed to do so.

These results suggest that neurotypical individuals can adapt their behavior according to the AI player and converge to an optimal game strategy by observing the sensorimotor patterns of their partner. In contrast, ASD patients seem to lack this ability, suggesting an impairment of socSMCs, possibly due to their lower predictive skills (Schmitz et al., 2003; Sinha et al., 2014; Wang et al., 2014).

### Discussion on Differences in Perception

To understand possible perceptual differences between the two groups, we looked at the questionnaires provided to the participants after the completion of each block, and the short structured interview at the end of the task. Participants at the end of each block reported how they evaluated the task, the target, and the other player in terms of engagement, predictability, and collaboration. Although we did not find any statistical significance in any of the items, participants in the ASD group seemed to perceive the task as overall more engaging than the TD group. Participants in the TD group perceived the task as less engaging when interacting with the “Wider” agent. When evaluating the agent’s predictability, the “Narrower” agent was perceived as less predictable by the ASD group compared to the TD. Playing with the different agents did not seem to affect target predictability in the TD group. However, we observe higher variability in the reported target predictability in the ASD group when playing with the “Narrower” and “Middle” agents. In terms of collaboration, both groups rated the “Middle” agent as more collaborative than the “Narrower” and “Wider.” Despite a lack of significance, our results provide possible insights on perceptual differences regarding the tasks’ characteristics with respect to the agent’s behavior. However, more data needs to be collected.

At the end of the task, we asked participants to report whether they thought they interacted with another human or a computer. We found no significant differences between the two groups. The agent was perceived as significantly more collaborative by participants that thought they were playing with a human instead of a computer. More specifically, the “Middle” agent was rated 50% more collaborative when participants thought it was another human. Indeed, according to Turing’s test (Turing, 1950), the behavior of a machine can be confused with that of a human.

Finally, the short-structured interview allowed us to assess further the perceived differences of the agents between the ASD and TD groups. The main differences arise from the characteristics used to describe the agents. Participants in the ASD group mainly commented on the agents’ movements, followed by their field use. The agents’ color was the characteristic less commented about. In contrast, the TD group differentiated between the agents by almost equally exploiting all three characteristics. The focus on movement as the main differentiating characteristic is something to be expected from the ASD group, as individuals in the spectrum tend to focus on moving objects. Moreover, the fact that almost no subject in the ASD group commented on the agents’ color as a significant trait could support the idea of the lack of model generation. If the agent’s color was a characteristic that could help participants predict its behavior, it would be unnecessary to consider it if no model was being created.

## General Discussion

The contributions of this study are two-fold. On the one hand, we formulated and introduced the Point of Social Subjective Equality (PSSE), a concept that allowed us to model the behavior of both humans and artificial agents in a collaborative task. By observing the PSSE, we quantified the degree of behavioral adaptation and how it can be modulated based on the variation of sensorimotor contingencies of the synthetic agent. On the other hand, this study demonstrated how collaborative behavior could implicitly emerge and be modulated through the observation of sensorimotor patterns of the partner.

Our behavioral analysis showed lower and slower adaptation to the artificial player by the ASD group. Similar results were found in Lieder et al. (2019), where participants in the autistic spectrum showed lower and slower adaptability in the task than their neurotypical counterparts. However, previous studies examining sensorimotor planning in individuals with ASD have yielded conflicting results. Some studies indicate an impairment in prospective control in ASD (Hughes, 1996; Scharoun and Bryden, 2016). In contrast, other studies showed no significant differences (Hamilton et al., 2007; van Swieten et al., 2010).

The larger variability in behavioral results of the ASD group is also present in the self-reported data. Nevertheless, the perceived predictability and collaboration during the task showed no differences between groups in these measures. Interestingly, the differences in the behavioral data but not in the self-reports raise the question of self-awareness. Could that be due to a lack of metacognition or due to a coping mechanism? Unfortunately, our current data do not allow us to answer this question, and further studies need to be conducted.

## Conclusion and Further Steps

In conclusion, this study adds to the literature possible ways of measuring collaboration through sensorimotor contingencies, and how this collaboration is impaired in individuals with ASD. While this study provides a preliminary insight, several limitations need to be discussed. First, further studies with larger sample sizes are needed to better control for individual differences. Furthermore, it is important to note that our study lacks female participants, as the main general users of the ASD center we collaborated with were males. This is in line with the larger occurrence of autism in male individuals compared to female ones. Despite these limitations, this study proposes a simple (and non-invasive) method to evaluate the predictive abilities of individuals in the autism spectrum. To do so, more data would be needed, as the main limitation of this study is the weakness of its statistical power.

Possible uses of this application would go in the line of an environment where the user could train their social abilities in a controlled and adaptive way. The system could be used to improve the abilities of non-neurotypical people by training their predictive skills. Like this, individuals in the spectrum could not only train their tracking of moving objects and predict their trajectories but also train their reading and understanding of non-verbal cues. The task offers the possibility of merging these two types of prediction (related to objects and social interaction), in a game-like manner.

To the moment, the PSSE has not been contrasted with any kind of diagnostic tool for ASD. In the future, a validation of the PSSE measurement in comparison with a screening tool could allow for a stronger claim on distinguishing between these two groups. However, at this point, we do not claim that it can be either a diagnostic tool or a tool to be able to distinguish between the two groups, but we highlight the possibility.

## Data Availability Statement

The raw data supporting the conclusions of this article will be made available by the authors, without undue reservation, to any qualified researcher.

## Ethics Statement

The studies involving human participants were reviewed and approved by the Parc de Salut Del Mar. Written informed consent to participate in this study was provided by the participants’ legal guardian/next of kin.

## Author Contributions

MB ran the experiments and data analysis, and wrote the manuscript. GM ran the PSSE code and data analysis. MS-F ran the PSSE idea and code. GM, MS-F, VV, and PV reviewed the manuscript. All authors read and approved the manuscript.

## Conflict of Interest

The authors declare that the research was conducted in the absence of any commercial or financial relationships that could be construed as a potential conflict of interest.

## Footnotes

1 https://scikit-learn.org/stable/modules/generated/sklearn.linear_model.LogisticRegression.html

2 https://fundaciofriends.org/es/servicios/educafriends/
